# Supplementary material for: Integrated multilayer stretchable printed circuit boards paving the way for deformable active matrix
Source: Nat Commun. 2019 Oct 28;10:4909. doi: 10.1038/s41467-019-12870-7 (PMC6817866; doi:10.1038/s41467-019-12870-7)
Supplement: Supplementary file 1 — Supplementary Information [file 41467_2019_12870_MOESM1_ESM.pdf]

Integrated Multilayer Stretchable Printed Circuit Boards Paving the way for Deformable Active Matrix

Biswas et al.

Supplementary Information

Supplementary Figures:

Supplementary Figure 1 **On-hard-carrier fabrication process of multilayer stretchable printed circuit boards**

Supplementary Figure 2 **Scanning electron microscope (SEM) image of a 10 µm thick Cu metal track.**

Supplementary Figure 3 ***Metal tracks crossing in the multilayer stretchable printed circuit board (SPCB).***

Supplementary Figure 4 **Influence of the descumming process.**

Supplementary Figure 5 **Vertical interconnect access (VIA) in the multilayer stretchable printed circuit board (SPCB).**

Supplementary Figure 6 **Solder coated pad.**

Supplementary Figure 7 **Scanning electron microscope (SEM) image of an array of Silicon based field effect transistors**

Supplementary Figure 8 **Encapsulation and the detachment process.**

Supplementary Figure 9 **Under-filling of EcoFlex.**

Supplementary Figure 10 **Active matrix in the stretchable substrate just after the peeling process.**

Supplementary Figure 11 **Stress profile of vertical interconnect accesses (VIAs) at different locations and dimensions.**

Supplementary Figure 12 **Peeling off the metal tracks and short circuit.**

Supplementary Figure 13 **3D guided deformations.**

Supplementary Figure 14 **Addressable LED array.**

Supplemental Figures:

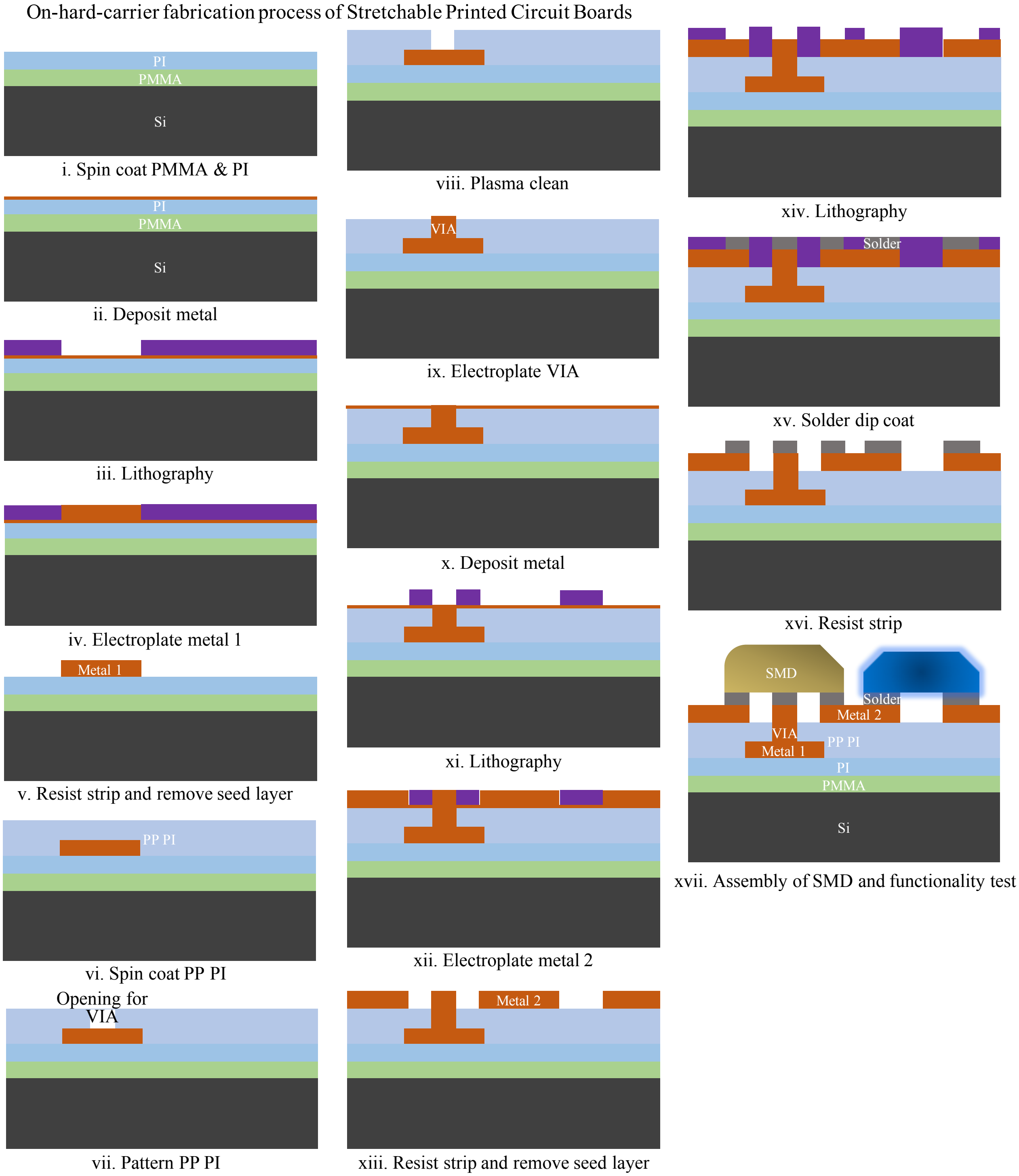

Supplementary Figure 1 On-hard-carrier fabrication process of multilayer stretchable printed circuit boards *using conventional planar microfabrication method. PMMA: poly(methyl-methacrylate), PI: polyimide, VIA: vertical interconnect access, SMD: surface mount device*

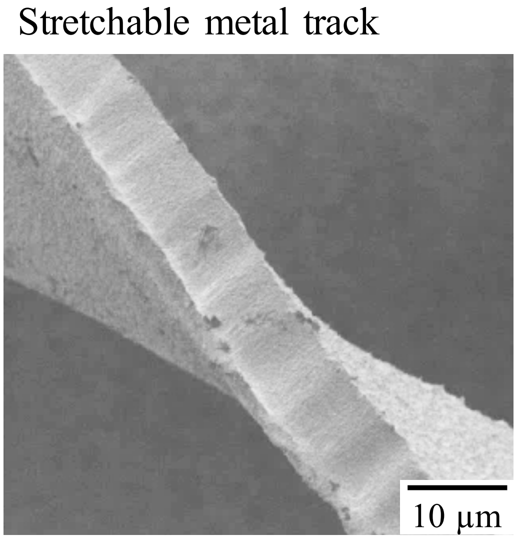

Supplementary Figure 2 **Scanning electron microscope (SEM) image of a 10 µm thick Cu metal track.**

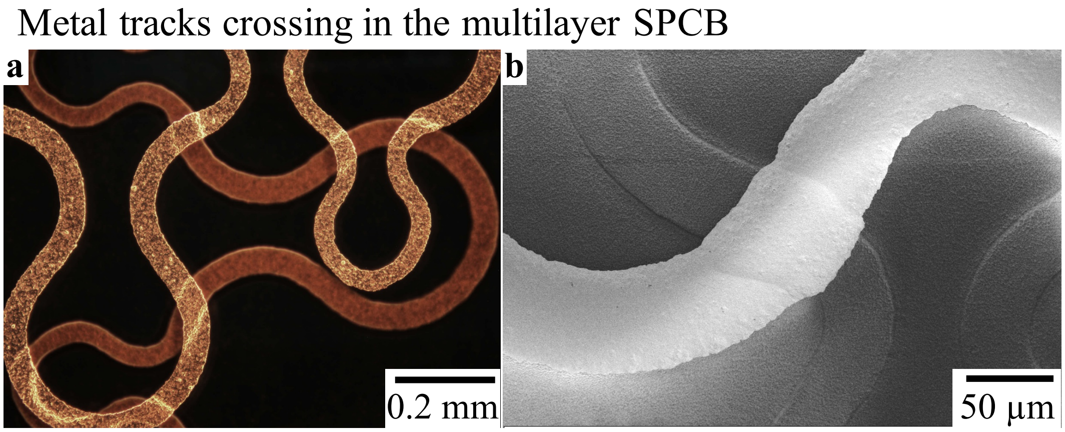

Supplementary Figure 3 ***Metal tracks crossing in the multilayer stretchable printed circuit board (SPCB).*** *An Optical microscope image (**a**) and a SEM image (**b**) of crossing regions of the multilayer metal tracks.*

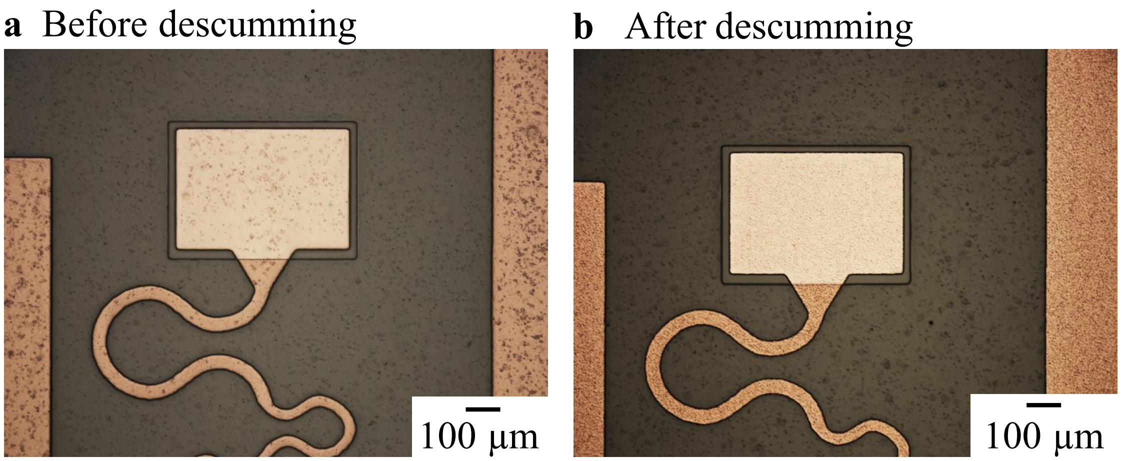

Supplementary Figure 4 ***Influence of the descumming process.*** *Optical microscope images showing the effect of descumming process prior to the VIA growing. The residues from photo-patternable PI (**a**) is removed by the descumming process (**b**), which improves the subsequent processes and the device performance.*

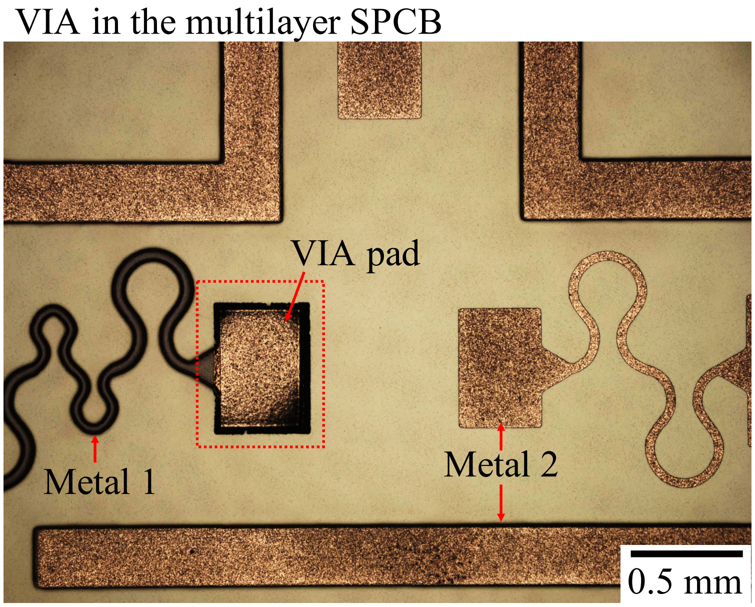

Supplementary Figure 5 ***Vertical interconnect access (VIA) in the multilayer stretchable printed circuit board (SPCB).*** *Optical microscope image of the multilayer metal tracks showing the VIA pad in the SPCB.*

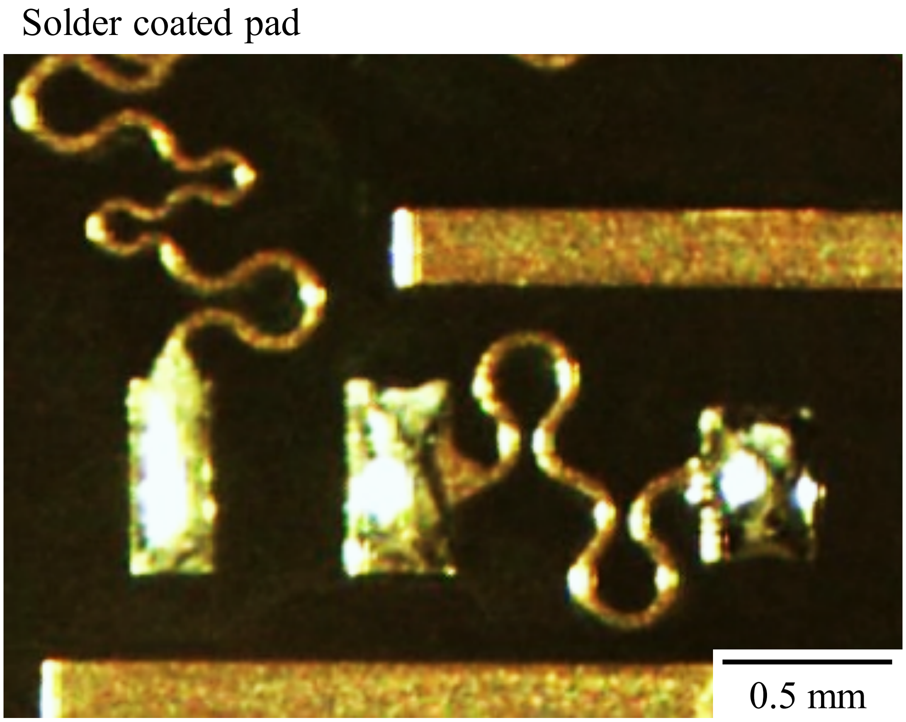

Supplementary Figure 6 **Solder coated pad.** *Optical microscope image of solder coated pads to assemble the surface mount components.*

Si based field effect transistors

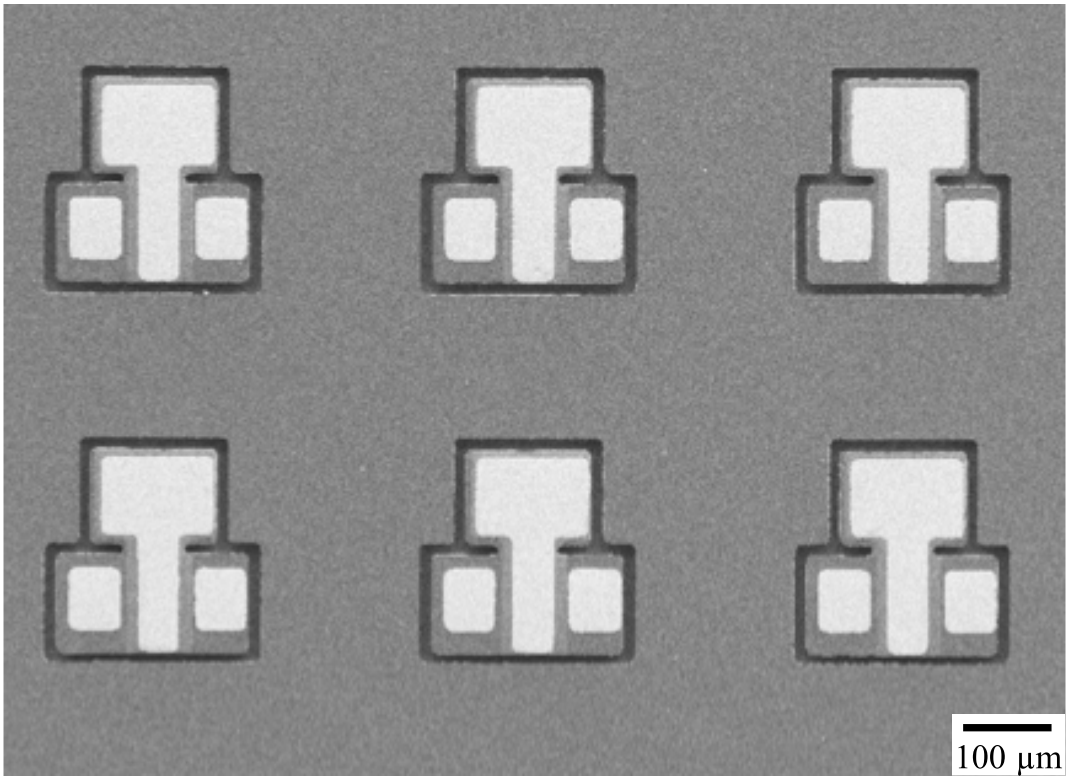

Supplementary Figure 7 ***Scanning electron microscope (SEM) image of an array of Silicon based field effect transistors*** *fabricated using spin-on-dopant.*

Encapsulation and the detachment process

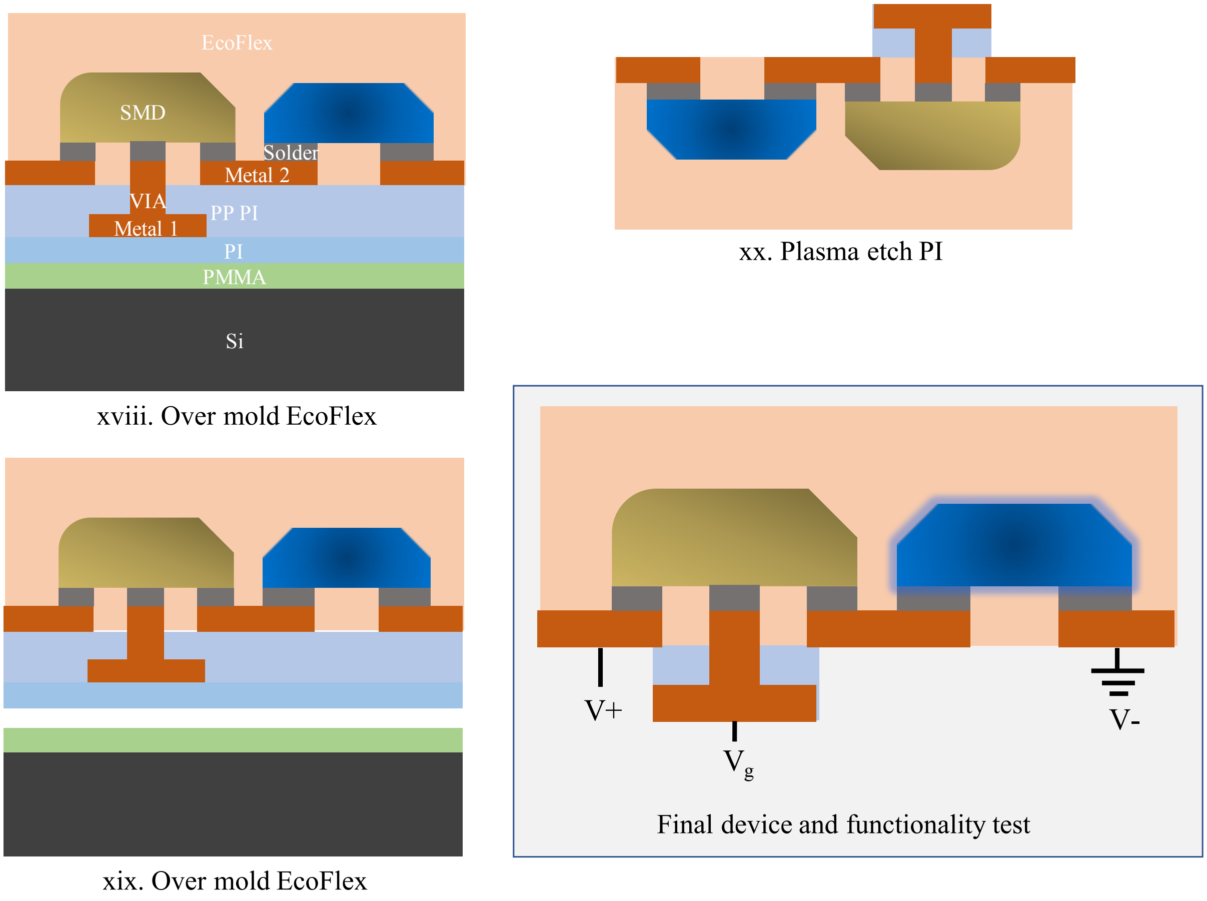

***Supplementary Figure 8* Encapsulation and the detachment process.** *Silicone encapsulation and the detachment process of the multilayer stretchable printed circuit boards from hard carrier. Insert shows the final device encapsulated in EcoFlex and test set up. PI: polyimide*

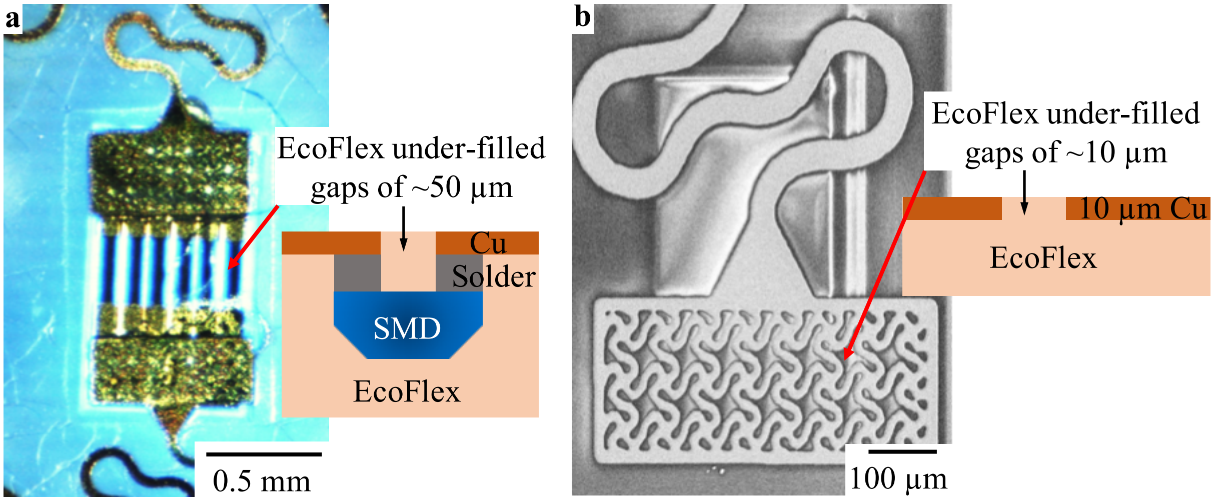

Supplementary Figure 9 **Under-filling of EcoFlex.** *EcoFlex 00-30 can under-fill very small gaps. (**a**) A back view of a LED embedded in EcoFlex showing the under-filling of EcoFlex in a gap of approximately 50 µm (schematic) and (**b**) a SEM image of a test structure showing that EcoFlex under-filled gaps of less than 10 µm (schematic).*

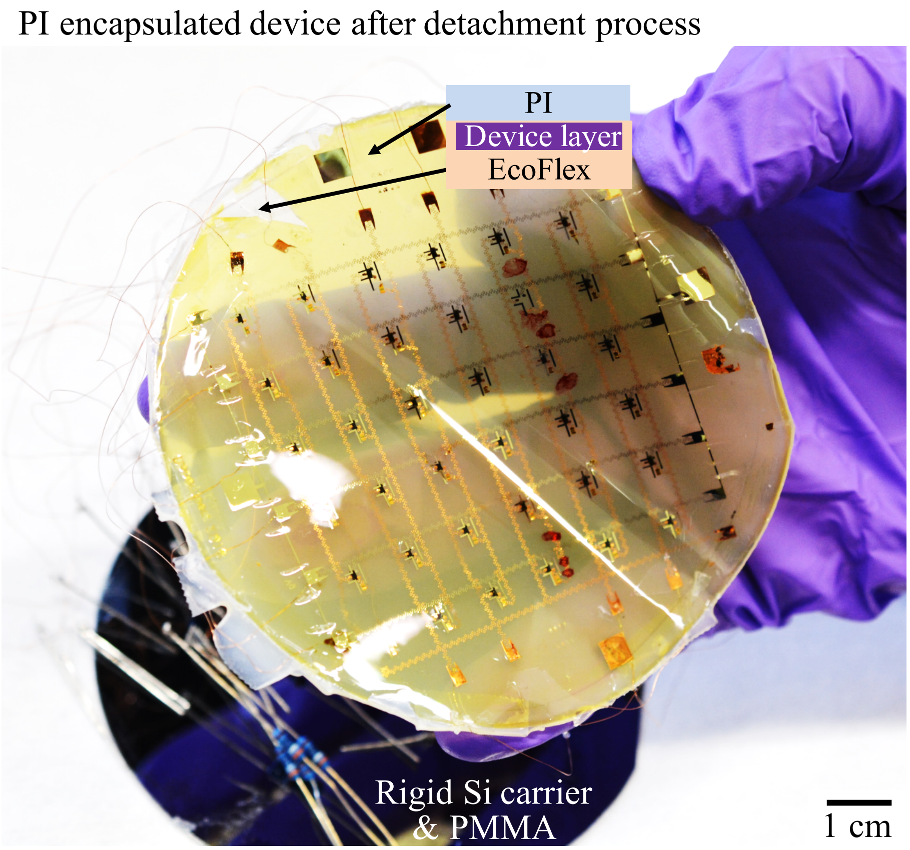

5 ***Supplementary Figure 10*** ***Active matrix in the stretchable substrate just after the peeling process.*** *The metal tracks remain covered with the PI (yellow layer) peeling layer which is later removed using plasma process.*

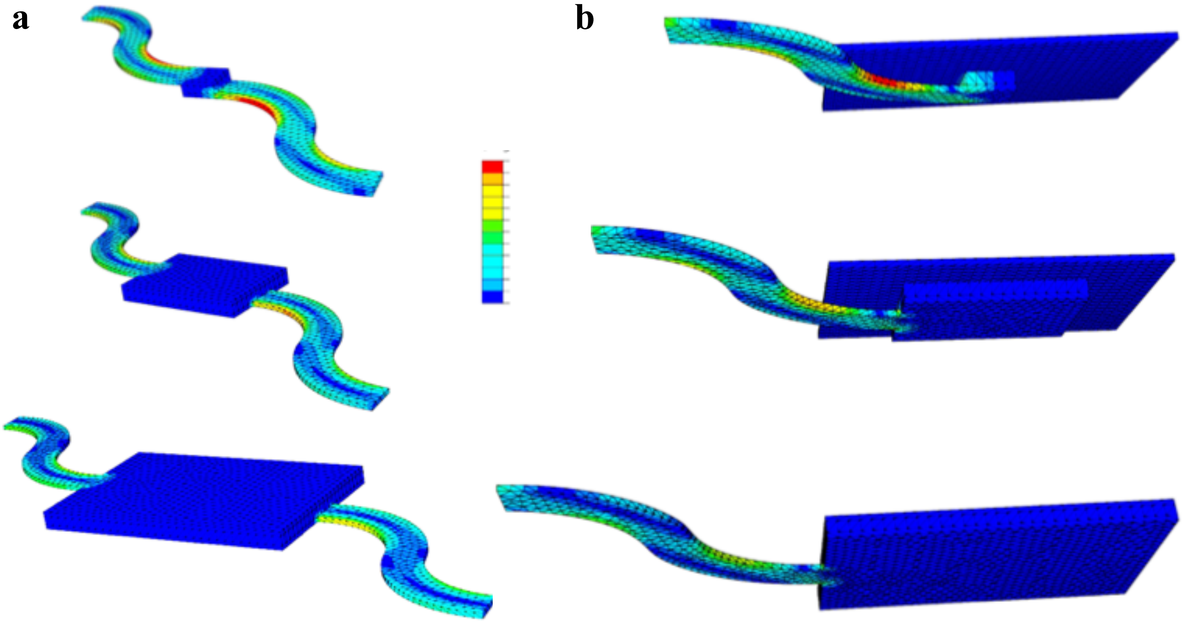

5 ***Supplementary Figure 11*** ***Stress profile of vertical interconnect accesses (VIAs) at different locations and dimensions.*** *(**a**) VIAs connecting bottom and top metal track in an open location and (**b**) VIAs connecting a metal track to one of the contact pads of a component.*

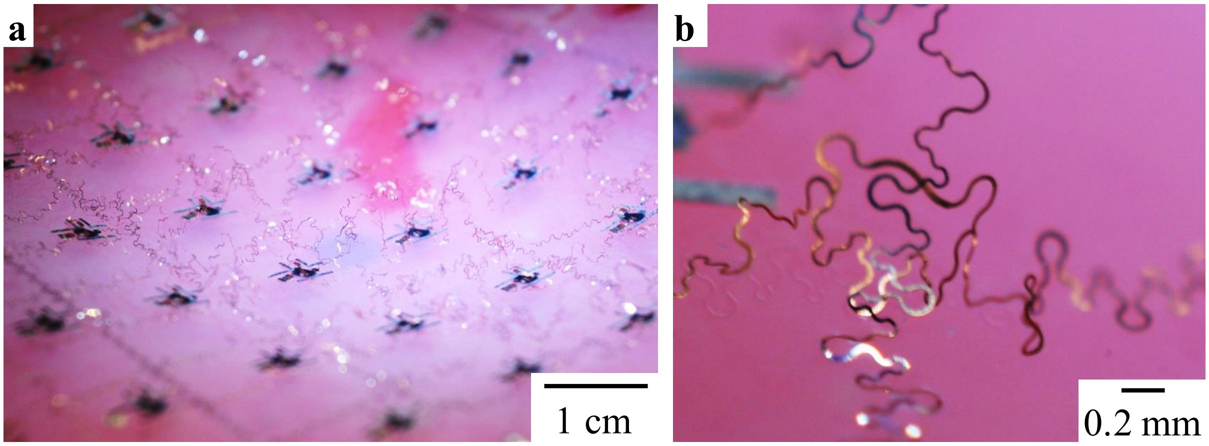

***Supplementary Figure 12* Peeling off the metal tracks and short circuit.** *Images of the detached metal tracks during stretching test. The metal tracks start to peel off from the rubber substrate and deformed randomly which eventually leads to short circuit.*

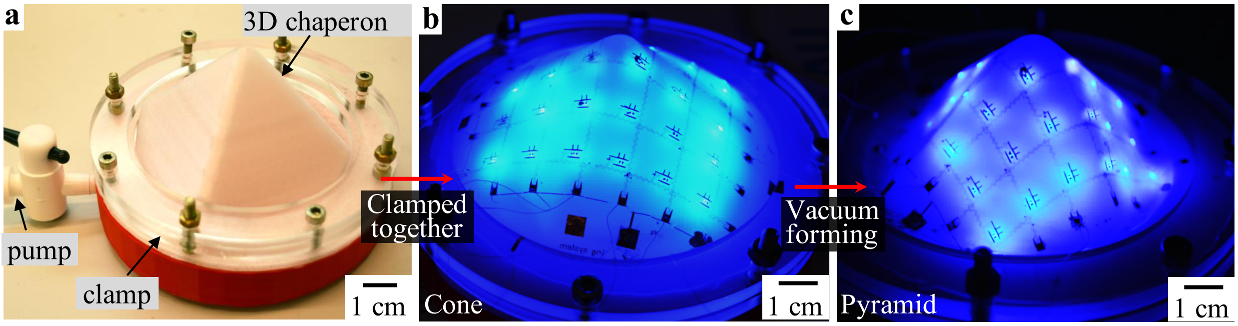

Supplementary Figure 13 ***3D guided deformations.*** *A 3D printed chaperon (**a**) is used to form a cone (**b**) which is further deformed to form a pyramid (**c**) through vacuum forming.*

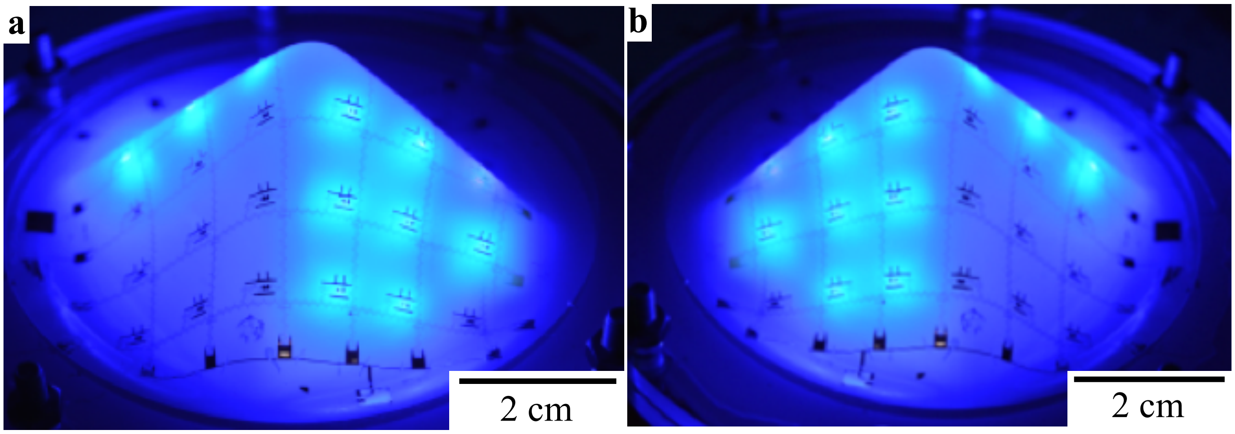

Supplementary Figure 14 ***Addressable LED array.*** *The deformed LED array remains fully addressable. Here two sides of the pyramid shape LED array are addressed subsequently (**a** & **b**).*

Supplementary Methods

Fabrication of Multilayer Integrated Stretchable Printed Circuit Board (SPCB) on Hard Carrier

Cleaning: A 500 µm thick Si wafer (MicroChemicals, Ulm, Germany) was cleaned in H_2SO_4 + H_2O_2 for 15 minutes and rinsed with DI water.

PMMA coating: A thick layer of poly(methyl-methacrylate) (PMMA) AR-P 6510 (Allresist, Strausberg, Germany) was spin coated at 1500 rpm and baked on a hot plate for 3 minutes at 180 °C

PI coating: A 8 µm thick polyimide PI 2611 (HD Microsystem, Neu-Isenburg, Germany) was spin coated at 1500 rpm and prebaked at 150 °C for 10 minutes on a hotplate and later fully cured in convection oven at 200 °C for 5 hours under N_2 flow.

Plasma activation: For plasma activation of the PI surface to increase the surface adhesion of the metal film to the PI, we used 30 SCCM O_2, 100 W RF power for 2 minutes.

Metallization: 50 nm/200 nm of Al/Cu was sputter deposited on top of plasma-activated PI layer.

Lithography: The wafer was then patterned for electroplating by photolithography using the negative resist AZ 15NXT (MicroChemicals, Ulm, Germany).

Descumming: A subsequent descumming process (2 minutes, 50 W, 50 SCCM O_2) was performed to remove any residues from the patterned resist.

Electroplating: A 10 µm thick layer of Cu was electroplated on top of the Cu seed layer using Cu 100 electrolyte (NB Technologies, Bremen, Germany). A current density of ~15 mA/cm^2 was applied to grow a smooth Cu layer at room temperature.

Resist strip: The negative resist was stripped using the NI555 remover at 80 °C.

Chemical etching: A chemical etching process was carried out to etch the 50 nm/200 nm Al/Cu seed layer using a Cu and Al etchant (MicroChemicals, Ulm, Germany).

Lithography: A 20 µm thick photo-patternable polyimide HD 4100 (HD Microsystem, Neu-Isenburg, Germany) was spin coated at 1500 rpm, baked at 150 °C for 10 minutes on a hotplate, and patterned for VIAs by photolithography.

Descumming: A subsequent descumming process (5 minutes, 50 W, 50 SCCM O_2) was performed to remove any residues from the patterned photo-patternable PI.

Electroplating: A 20 µm tall VIA of Cu was electroplated at the openings using the first metal tracks as the seed layer.

Cleaning: The wafer was dipped into 5% HCl and in H_2SO_4 + H_2O_2 for 1 minute and rinsed with DI water.

Plasma activation: The photo-patternable PI surface was plasma activated to increase the surface adhesion, we used 30 SCCM O_2, 100 W RF power for 2 minutes.

Metallization: 20 nm/200 nm of Ti/Cu was sputter deposited as a second metallization layer.

Lithography: The wafer was then patterned for electroplating by photolithography using the negative resist AZ 15NXT.

Electroplating: Another 10 µm thick layer of Cu was electroplated on top of the Cu seed layer.

Resist strip: The negative resist was stripped using a remover.

Chemical etching: A chemical etching process was carried out to etch the 20 nm/200 nm Ti/Cu seed layer.

Lithography: The wafer was then patterned for soldering by photolithography using the positive resist AZ 1518 and baked at 120 °C for 10 minutes.

Soldering: The pads were coated by dip coating in a solder bath (Indalloy #117, MP. 47 °C, Indium Corp., NY).

Resist strip: The resist was stripped using a remover.

Rinsing: The sample was finally rinsed with DI water and dried under N_2.

Assembly of the SMD components: The SMD components were assembled on the wafer following a standard pick-and-place technique. The wafer was heated from the back to melt the

solder and then the surface mounted components were assembled. The reinforcement frames were assembled subsequently. Device tests were performed on the wafer to check the interconnections and the device performance was compared before and after the detachment process.

Over molding EcoFlex: The silicone EcoFlex (Smooth-On, EcoFlex 00-30) mold was prepared by mixing Part A and Part B (1:1 volume ratio) and by degassing the mixture in a desiccator. The liquid mold of silicone was poured on top of the substrate with assembled components and cured overnight at room temperature.

Detachment: First, EcoFlex was removed manually from the edges of the wafer as EcoFlex is strongly adhesive to bare Si. Then the EcoFlex layer was peeled using the sacrificial PI layer.

Dry etching of the sacrificial PI: As a final step, the sacrificial PI peeling layer was etched in ECR (40 SCCM O_2 + 10 SCCM CF_4, 100 W RF power, 0.025 mbar) for 30 minutes.

Fabrication of the Si Transistors

Doping: The transistors were fabricated using spin-on-dopant (SOD). 500 µm thick p-type Si wafer (MicroChemicals, Ulm, Germany) with resistivity of 10-20 ohm-cm was cleaned in H_2SO_4 + H_2O_2 for 15 minutes and subsequently rinsed with DI water. For masking, a 500 nm SiO_2 layer was grown using a wet oxidation process at 1050 °C (Tempress). The oxide was patterned by photolithography using AZ 1518 photoresist and etching in HF before the resist was removed. A subsequent piranha (H_2SO_4 + H_2O_2) cleaning process was performed. Next n-type SOD (Filmtronics, P509) was spin coated at 1500 rpm for 60 seconds and baked at 250 °C for 30 minutes. The annealing process was performed at 1050 °C under N_2 flow (400 SCCM) for one hour. Later SOD residues and SiO_2 mask were etched in HF for 8 minutes to remove everything from the surface. Alignment marks for the source and drain were covered manually with the photoresist before the etching process was performed, and the resist mask was removed after the etching. Later a cleaning process was performed.

Gate Oxide: On the clean surface high quality 100 nm dry oxide was grown for the gate oxide at 1000 °C and patterned using photolithography and later etched in HF. The resist was removed in remover AZ100 and the wafer was washed in DI water and dried.

Metallization: The metallization layer was fabricated by photolithography and lift off. We used negative AZ NXT15 resist and 2 minutes of descumming to remove any residues. E-beam evaporation was used to coat the surface with 100 nm Al + 50 nm Pt + 200 nm Au. Lift-off was done in Dimethylsulfoxide (DMSO) overnight. A rapid thermal process (RTP) process was performed at 400 °C for one minute.
